# Liver Transplantation Using a Whole-Liver Graft Donated from a Brain-Dead Donor Complicated with Rendu–Osler–Weber Syndrome: A Case Report

**DOI:** 10.70352/scrj.cr.25-0277

**Published:** 2025-09-03

**Authors:** Takuro Fujita, Akihiko Soyama, Taiichiro Kosaka, Takanobu Hara, Hajime Matsushima, Ayaka Kinoshita, Hajime Imamura, Takashi Hamada, Akaya Satoh, Kazushige Migita, Yuta Kawaguchi, Tomohiko Adachi, Susumu Eguchi

**Affiliations:** Department of Surgery, Nagasaki University Graduate School of Biomedical Sciences, Nagasaki, Nagasaki, Japan

**Keywords:** Osler–Weber–Rendu syndrome, hereditary hemorrhagic telangiectasia, brain-dead donor, extended-criteria donor

## Abstract

**INTRODUCTION:**

Osler–Weber–Rendu syndrome, or hereditary hemorrhagic telangiectasia (HHT), is a rare autosomal dominant genetic vascular disease characterized by arteriovenous malformations, vascular wall fragility, dilatation, and rupture of the vessels with hepatic symptoms. As HHT with hepatic symptoms is recognized as the primary etiology for liver transplantation, liver transplantation with liver grafts from donors affected by HHT is extremely rare. Herein, we report a successful liver transplantation in a patient with biliary atresia who received a whole-liver graft from a young brain-dead donor with HHT.

**CASE PRESENTATION:**

The patient was a 15-year-old girl with decompensated liver cirrhosis who underwent Kasai surgery for biliary atresia at 3 months of age. The donor was a female in her teens, diagnosed with brain death due to cerebral hemorrhage. Although the donor was diagnosed with Osler disease, she had no hepatic symptoms and normal liver function. CT did not reveal any apparent vascular malformations in the liver. A whole-liver transplant was performed using the donated liver. The patient recovered well in terms of liver function, without any hepatic-related symptoms.

**CONCLUSIONS:**

Our experience with this patient may have important implications for liver transplantations from donors with HHT.

## Abbreviations

AP shunt
: arterioportal shunt
AV shunt
: arteriovenous shunt
Hb
: hemoglobin
HHT
: hereditary hemorrhagic telangiectasia
MELD
: Model for End-Stage Liver Disease
VM
: vascular malformation

## INTRODUCTION

The imbalance between donors and patients on the waiting list for liver transplantation is a persistent problem worldwide. Although the number of organ donations is gradually increasing in Japan, the number remains significantly lower than that of other countries.^1,2)^ To improve this situation, liver transplantation with liver grafts from extended-criteria donors has been performed with reasonable posttransplant outcomes.^3)^ Besides the widely recognized risk factors such as donor age, steatosis, donation after cardiac death, and long ischemic time, a liver graft donated from a donor complicated with a systemic disease that might affect liver function is usually considered an extended-criteria donor graft.

Osler–Weber–Rendu syndrome, or HHT, is a rare autosomal dominant genetic vascular disease characterized by arteriovenous malformations and pathological angiogenesis. This causes vascular wall fragility and dilatation, and the consequent rupture of the vessels in vital organs, including the brain, lungs, and liver.^4)^ Because HHT is considered an indication for liver transplantation, liver transplantation with liver grafts from donors with HHT is extremely rare. To the best of our knowledge, only one such case has been reported.^5)^

Herein, we report a successful case of liver transplantation in a patient with biliary atresia who received a whole-liver graft from a young brain-dead donor affected by HHT.

## CASE PRESENTATION

Ethical approval for this case report was obtained from the Institutional Review Board of Nagasaki University Hospital (No. 19102143) on October 22, 2019. This study was performed in line with the SCARE (Surgical CAse REport) criteria.^6)^

The patient was a 15-year-old girl who underwent Kasai surgery for biliary atresia at 3 months of age. At 6 years of age, esophageal varices due to portal hypertension were detected. At 8 years of age, she underwent laparoscopic splenectomy due to gastrointestinal bleeding caused by ruptured varices. At the age of 14 years, she experienced a critical rupture of varices with a Hb level of 2.8 mg/dL and was diagnosed with hepatopulmonary syndrome associated with a portal vein shunt. The patient was then referred to our department for liver transplantation. She was 148 cm tall, weighed 35.2 kg, and her blood and biochemical tests at the time of referral were as follows: platelets: 170000/μL; prothrombin time: 93%; albumin: 2.4 g/dL; creatinine: 0.39 mg/dL; total bilirubin: 5.2 mg/dL; direct bilirubin: 4.1 mg/dL; aspartate aminotransferase: 138 U/L; alanine aminotransferase: 83 U/L; γ-glutamyl transpeptidase: 149 U/L; Child–Pugh classification: C; MELD score: 10 points. When the patient experienced variceal bleeding, her Hb level critically fell to 2.0 mg/dL. The esophageal varices were classified as F3, with a positive red color sign, indicating a constant risk of rebleeding. This situation necessitated urgent intervention for portal hypertension. The combination of severe factors, including the potential for critical bleeding and hypoxemia due to hepatopulmonary syndrome, led us to consider that liver transplant was indicated. After the application was submitted, the indication was approved by the indication evaluation committee.

**Fig. 1:**
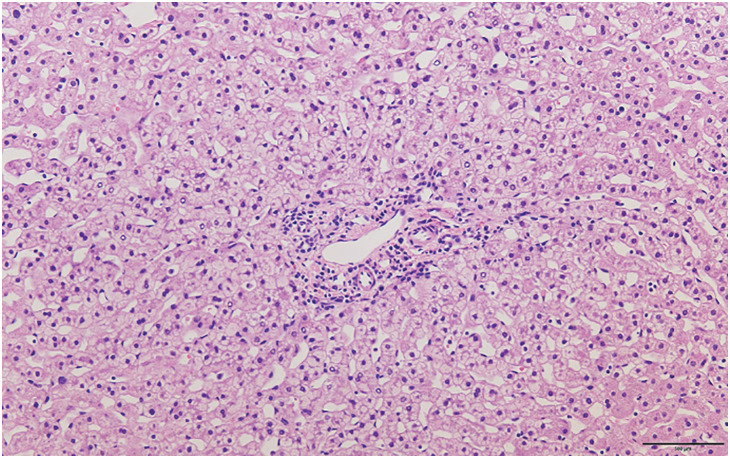
Microscopic findings of time-zero biopsy.

During her registration on the brain-dead liver transplantation waiting list, the following donor information was provided: The donor was a female in her teens, with a height of 138 cm and a weight of 29.7 kg, who suffered brain death due to cerebral hemorrhage. She had a family history of HHT, and the donor was diagnosed with Osler’s disease, which was confirmed by genetic testing after the cerebral hemorrhage. The donor’s liver function was normal, and although a possible intrahepatic partial AP shunt was suspected on imaging, it was not clear whether the finding was due to Osler’s disease. Since no liver function problems were observed, we decided to conduct a whole-liver transplant using the liver donated by the donor. The surgical findings were as follows:

During laparotomy, significant adhesions were found due to a previous surgery. After total hepatectomy, the entire liver was transplanted using the piggyback technique. The operation duration was 12 h 49 min, with an estimated blood loss of 5404 g. The total ischemic time inhibition was 9 h 33 min, the graft liver weight was 614 g, and the standard graft weight/recipient liver volume was 66.9%. Time-zero biopsy showed no abnormal findings, such as fibrosis, inflammation, or vascular lesions (**Fig. 1**). Postoperatively, the patient’s respiratory and circulatory status was stable, and immunosuppression with tacrolimus and steroids was initiated on POD 1. The patient was extubated, and on POD 4, the patient was discharged from the ICU and managed in the general ward. Postoperatively, the patient recovered well in terms of liver function and general condition, but on the 10th day, there was an increase in the inflammatory reaction, and on the 11th day after surgery, emergency surgery was performed due to a diagnosis of perforation at the site of the repaired colonic wall due to adhesiolysis during transplantation.

**Fig. 2:**
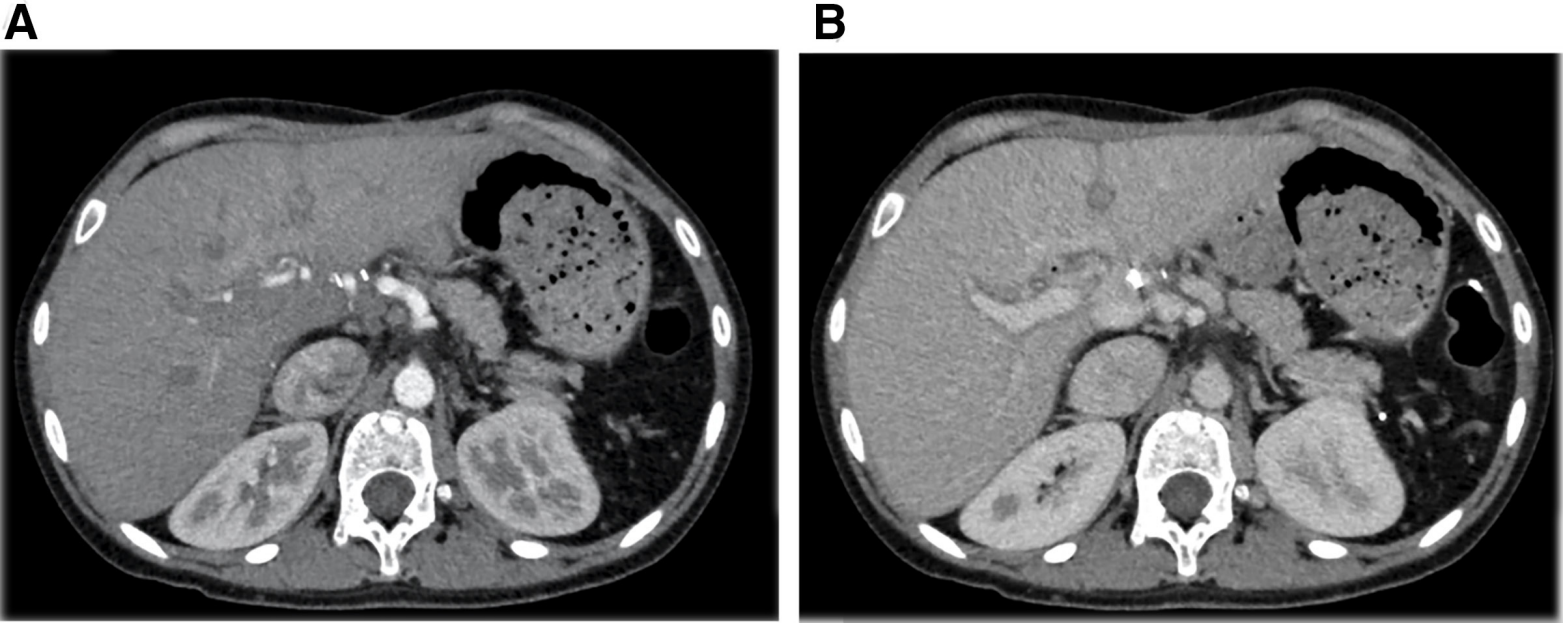
Postoperative CT scan at 3 months after transplantation showing homogeneous enhancement without apparent vascular malformation. (**A**) Arterial phase and (**B**) portal phase.

Postoperatively, in addition to the colon perforation, she developed a subarachnoid hemorrhage event associated with reversible cerebral vasoconstriction syndrome. The intracranial event in this patient was associated with reversible cerebral vasospasm syndrome, indicating it was not connected to the liver graft from the donor with HHT. Otherwise, her liver function and general condition showed good recovery, and she was discharged 50 days after the transplant. Contrast-enhanced CT performed 3 months after transplantation showed good hepatic perfusion, homogeneous enhancement, and no apparent VMs associated with HHT (**Fig. 2**). During outpatient follow-up, acute steroid-resistant rejection was observed that improved with thymoglobulin. Before the transplantation, the patient experienced hypoxemia, with an SpO_2_ of 88% and a PO_2_ of 48.2 mmHg while breathing room air. Consequently, home oxygen therapy was initiated. Following the liver transplant, her SpO_2_ improved to approximately 98% on room air, eliminating the need for supplemental oxygen. When this case report was written, with a follow-up 1 year after transplantation, the patient’s liver function was normal, and there were no complications related to the transplant.

## DISCUSSION

We report a successful liver transplantation using a whole-liver graft from a donor diagnosed with Osler–Weber–Rendu syndrome. Gruttadauria et al. reported the first case of liver transplantation using a whole graft from a deceased donor affected with Osler–Weber–Rendu syndrome.^5)^ Besides liver transplantation, a previous study reported a successful kidney transplantation from a donor with Rendu–Osler–Weber syndrome who died secondary to a polymicrobial brain abscess.^7)^

HHT is the primary etiology for liver transplantation.^8,9)^ Hepatic arteriovenous malformations are estimated to occur in 70% of patients with HHT and are more common in women than in men. Hepatic arteriovenous malformations include hepatic artery–portal vein shunts (AP shunt), hepatic artery–hepatic vein shunts (AV shunt), and portal vein–hepatic vein shunts (portosystemic shunt). Although AP shunts are the most common among the 3 types of shunts, they may be mixed. Symptomatic cases include heart failure, biliary necrosis, and portal hypertension due to a large number of shunts. Heart failure is the most common condition, whereas the latter 2 are less common. Patients with symptoms of hepatic VMs (including high-output cardiac failure) who do not respond to intensive medical management are indicated for liver transplantation.

Therefore, transplant surgeons may be reluctant to consider liver transplantation using grafts from donors affected by HHT. However, transplant surgeons should recognize the risk of hepatic manifestations caused by HHT. Although liver involvement is common in HHT, most patients do not present with any hepatic symptoms. Hepatic VMs are observed in up to 74% of patients with HHT, but only 8% are symptomatic at diagnosis.^10)^ The mean age at diagnosis of liver VMs is 48 years, and the condition is more common in females.^11,12)^

The donor in this case had no apparent hepatic VMs due to HHT and had good liver function. The donor was in her teens, markedly younger than the age at which HHT-induced liver symptoms are most likely to occur. After considering both the recipient’s condition and the donor’s risk of future liver symptoms, we concluded that acceptance and transplantation of the donated liver would be beneficial to the recipient’s life expectancy.

Liver transplantation using a diseased liver, such as domino transplantation from a patient with familial amyloid polyneuropathy, maple syrup urine disease, or familial hypercholesterolemia, is considered an important therapeutic strategy to increase the number of available liver grafts for patients.^13,14)^ Furthermore, to overcome the difficulties encountered in obtaining liver grafts for specific categories of patients, such as neonates who urgently need a liver transplant, pediatric domino transplant with a left-lateral segment of a metabolically abnormal liver obtained from a 7-year-old patient with primary oxalosis as bridging therapy to allow sufficient growth to permit a definitive liver transplantation, was reported.^15)^

Although the liver in this case was donated from a patient with Osler’s disease, the liver itself was not diseased at the time of transplantation. It is important to carefully follow the possible impact of Osler’s disease on the long-term status of the graft. Since there are very few reports on liver transplants from HHT donors, it is important to carefully monitor the possible impact of Osler’s disease on the long-term status of the graft.

## CONCLUSIONS

Our experience with this case may have important implications for the interpretation of liver transplantation in donors with systemic diseases affecting the liver.

## DECLARATIONS

### Availability of data and materials

The data supporting the findings of this study are available from the corresponding author upon reasonable request.
